# Qi Ling decreases paclitaxel resistance in the human prostate cancer by reversing tumor-associated macrophages function

**DOI:** 10.18632/aging.203904

**Published:** 2022-02-22

**Authors:** Hongwen Cao, Dan Wang, Renjie Gao, Yigeng Feng, Lei Chen

**Affiliations:** 1 Surgical Department I (Urology Department), Longhua Hospital Shanghai University of Traditional Chinese Medicine, Xuhui, Shanghai 200032, China

**Keywords:** Qi Ling, paclitaxel resistance, prostate cancer, tumor-associated macrophages

## Abstract

Tumor-associated macrophages (TAMs) are critical immune cells infiltrated into tumor. In present study, we evaluated the effects of Qi Ling (QL), a traditional Chinese medicine on paclitaxel resistance in prostate cancer cells and explored the underlying mechanisms. We administrated QL to rats and collected the serum from QL-treated rats (QL-serum). We established the co-culture system of TAMs/paclitaxel resistant prostate cancer cells. We treated the TAMs with QL-serum and measured the viability of paclitaxel resistant prostate cancer cells after exposing to paclitaxel. We monitored the expression of M1 and M2 markers, the expression and activation of IL-6/STAT3 signaling pathways in TAMs after QL treatment. We treated TAMs with QL-serum together with interleukin (IL)-6, measured the expression of M1 and M2 markers, and the viability of paclitaxel resistant prostate cancer cells. In co-culture system, QL-serum-treated TAMs decreased the paclitaxel resistance in the human prostate cancer cells. QL-serum treatment significantly up-regulated the expression of M1 markers inducible nitric oxide synthase and tumor necrosis factor α while decreased the expression of M2 markers IL-10 and chemokine (C-C motif) ligand 22. QL-serum suppressed the activation of IL-6/ signal transducer and activator of transcription 3 signaling pathway. All these effects of QL-serum were abolished in the presence of IL-6. Qi Ling re-programmed TAMs and decreases paclitaxel resistance in prostate cancer cells.

## INTRODUCTION

Prostate cancer, the cancer of the prostate, is one common cancer among men [1]. Prostate cancer is with high morbidity and mortality, which cause great burden over the world [2]. In 2010, the costs associated with prostate cancer diagnosis and treated was around 12 billion in United States, and the cost of prostate cancer treatment increase more quickly than any other cancer [3]. Androgen deprivation treatment is a common strategy to treat prostate cancer with great efficiency for over 80% prostate cancer. However, after several years 50% of the cases become resistant to this treatment. Recently, paclitaxel has been used to treat prostate cancer and has good efficacy [4]. Despite the clinical success of paclitaxel, the limitations of paclitaxel including acquisition of resistance are still needed to be resolved [5]. Understanding the underlying mechanisms of paclitaxel resistance could shed light on improving the efficiency of prostate cancer treatment.

Tumor-associated macrophages (TAMs) are essential factors in the tumor microenvironment (TME). TME is composed of multiple immune cells including T cells, dendritic cells and macrophages. TAMs are the most abundant cells in TME. Macrophages are usually classified into two distinct polarized phenotypes including M1 and M2 [6]. Lipopolysaccharides and type 1 T helper cell cytokines such as interleukin (IL)-12, IL-18 induce M1 polarization. M1 macrophages produce typical pro-inflammatory cytokines and chemokines and have antitumor effects [7]. In contrast, M2 polarized macrophages are induced by type 2 T helper cell cytokine such as IL-4 and IL-10 and produce anti-inflammatory including IL-10, transforming growth factor-beta (TGF-β) and IL-13. M2 macrophages promote tumor development and are considered as pro-tumor macrophages. The TAMs are believed to more closely resemble the M2 phenotype and display immunosuppressive and pro-tumorigenic function [8]. Increasing evidences have implicated the TAMs in cancer chemotherapy resistance. Inhibition of TAMs has been shown to attenuate chemotherapy resistance. TAMs secret IL-6, and IL-6/signal transducer and activator of transcription 3 (STAT3) signaling pathway mediates the resistance in tumor to chemotherapy drugs [9]. Herein, it may be effective for decreasing tumor chemotherapy resistance via targeting TAMs and related IL-6/STAT3 signaling pathways.

Qi Ling (QL) is a traditional Chinese medicine with potent benefits against prostate tumors. Clinical evidences have suggested beneficial effects of QL in improving symptoms and life quality, and prolonging overall survival. In present study, we evaluated the potential effects of QL on paclitaxel resistance in the prostate cancer and explored the underlying mechanisms.

## MATERIALS AND METHODS

### Cell culture

The paclitaxel resistant prostate cancer cells DU145-TxR and PC-3-TxR were cultured as described previously [10]. Phorbol-12-myristate-13-acetate (PMA, 100 ng/mL) (Sigma, St. Louis, MO, USA) was used to treat THP-1 cells for 12 h to generate macrophages. Then the TAMs were cultured on 0.4 μm pore inserts and then transferred to cell culture plate with DU145-TxR or PC-3-TxR.

### QL treatment

*QL* was composed of 150 g of raw astragalus, 300 g of rubescens, 150 g of cooked Rehmannia glutinosa, 150 g of psoraleae, 150 g of motherwort, 90 g of turmeric, 150 g of Kushan and 90 g of processed licorice. The mixture was boiled in H_2_O for 3 h and refilled to 1 L. 20 ml of QL was administered to Lewis male rats with weight of 180–220 g by oral gavaging twice a day for 7 consecutive days. One hour after the final gavage, blood was collected from the abdominal aorta, and serum was separated after centrifuge. After inactivation at 56°C for 30 min, the serum was filtered through 0.22 μm microporous membrane to get rid of bacteria, and stored at −80°C for later use. Since QL is a mixture of traditional Chinese medicine, all our experiments were carried out with the same batch of rat serum. 1% rat serum containing QL (QL serum) was added to TAMs cell culture medium for 24 h. Phosphate-buffered saline (PBS) was administrated to rats to obtain control serum (PBS serum). In certain experiment, 200 ng/mL of IL-6 was used to treat TAMs. Then different concentrations of paclitaxel were added to DU145-TxR or PC-3-TxR and incubate for another 24 h.

### Cell viability assay

Cell viability was assessed using 3-(4,5-Dimethylthiazol-2-yl)-2,5-diphenyltetrazolium bromide (MTT) assay kit (Abcam, Shanghai, China) following manufacture’s protocols 24 h after paclitaxel treatment.

### RT-PCR

RNA from TAMs was extracted using TRIzol reagent (Thermo Fisher, Waltham, MA, USA) following the manufacturer’s protocol. The cDNA was synthesized using PrimeScript™ RT Master Mix (Takara, Beijing, China). The real time PCR was performed using TB Green^®^ Advantage^®^ qPCR Premix (Takara) on QuantStudio 5 Real-Time PCR System. Primers used for real time PCR were listed as follows: inducible nitric oxide synthase (*iNOS)* Forward (F): GCCAAGCTGAAATTGAATGAGGA, Reverse (R): TTCTGTGCCGGCAGCTTTAAC. Tumor necrosis factor α (*TNF-α)* F: CTGTAGCCCATGTTGTAGCAAAC, R: GCTGGTTATCTCTCAGCTCCAC. *IL-10* F: TTTAAGGGTTACCTGGGTTGC, R: TTGATGTCTGGGTCTTGGTTC. Chemokine (C-C motif) ligand 22 (*CCL22*) F: TGCCGTGATTACGTCCGTTA, R: TCTCCTTATCCCTGAAGGTTAGCA. *IL-6* F: ACAGGGAGAGGGAGCGATAA, R: GAGAAGGCAACTGGACCGAA. *GAPDH* (internal control gene) primer F: GCACCGTCAAGGCTGAGAAC; R: TGGTGAAGACGCCAGTGGA.

### ELISA

Cytokine concentrations in cell culture supernatant or cell lysates were measured by ELISA kits (Abcam) following manufacturer’s instructions.

### Western blot

TAMs were lysed for protein extraction using cell lysis buffer (Abcam). The equal amount of protein was subjected to sodium dodecyl sulfate–polyacrylamide gel electrophoresis and then was transferred to polyvinylidene fluoride membrane. Following blocking in 5% non-fat milk for 1 h at room temperature, the membranes were incubated with primary antibodies at 4°C for overnight. Next day, after wash the membranes were incubated with corresponding horse radish peroxidase-conjugated secondary antibodies for 1 h at room temperature. All antibodies were purchased from Abcam. The immunoreactive bands were detected by adding the ECL Substrate (Abcam).

### Statistics analysis

The data were represented as mean ± standard deviation (SD). Two/one-way ANOVA with appropriate post hoc tests, and Student’s *t*-test were used to calculate *p* value. All experiments were repeated at least in triplicate. When *p* < 0.05 the difference was considered as significant.

## RESULTS

### TAMs treated with QL serum decreased paclitaxel resistance in prostate cancer cells

**Figure 1:**
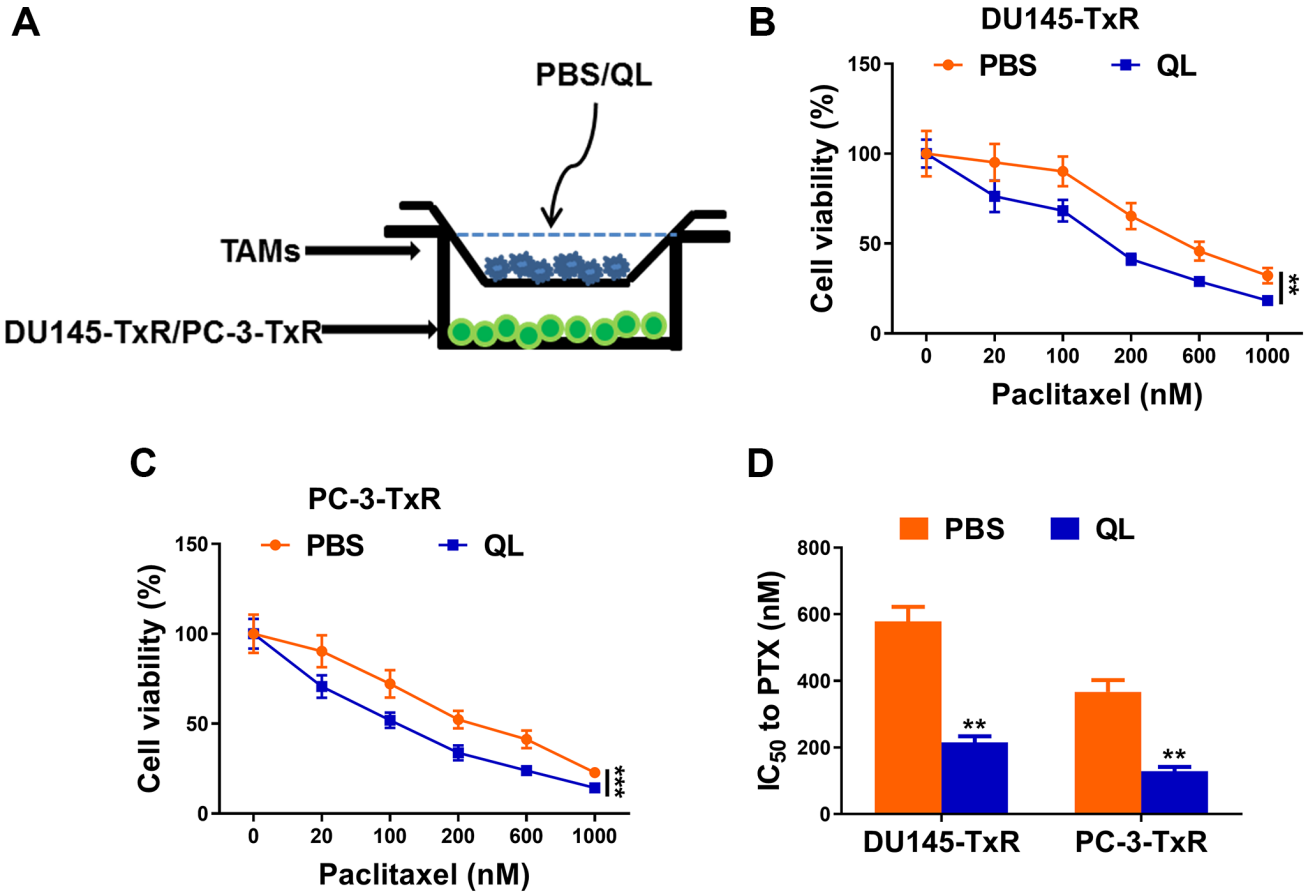
**Qi Ling serum-treated TAMs decreased paclitaxel resistance in prostate cancer cells.** (**A**) Schema for co-culture system of paclitaxel-resistant prostate cancer (DU145-TxR and PC-3-TxR) cells and tumor-associated macrophages (TAMs). (**B** and **C**) MTT assay showed viability of DU145-TxR and PC-3-TxR cells harvested from the co-culture system exposed with indicated concentrations of paclitaxel for 24 h. (**D**) IC50 values of DU145-TxR and PC-3-TxR cells harvested from the co-culture system were determined from the viability versus paclitaxel concentration curves. The data represent the mean ± SD. ^**^p < 0.01; ^***^p < 0.001, determined by two-way ANOVA followed a post hoc test for panels (**B** and **C**), Student’s *t*-test for panel (**D**).

To mimicking the real usage of QL in practice, we administrated QL to rats and harvested the serum for future usage. We analyzed the cytokine level of TNF-α, IL-1β and IL-6 in rat serum and found significantly increased TNF-α and decreased IL-6 in QL serum. The serum level of IL-1β was similar between QL treated rats and PBS-treated rats (Supplementary Figure 1). We established the co-culture system of TAM with paclitaxel resistant prostate cancer cells DU145-TxR or PC-3-TxR (Figure 1A) and treated TAMs with QL serum. The cell viability of DU145-TxR and PC-3-TxR were measured after exposure to difference concentration of paclitaxel. As shown in Figure 1B, the cell viability of DU145-TxR decreased after paclitaxel treatment in a dose-dependent manner. At each concentration of paclitaxel, the viability of DU145-TxR co-cultured with QL serum-treated TAMs was significantly decreased when compared DU145-TxR co-cultured with PBS serum-treated TAMs, indicating QL serum-treated TAMs decreased the paclitaxel resistance in DU145-TxR. Similarly, PC-3-TxR co-cultured with QL serum-treated TAMs had remarkably lower viability than PC-3-TxR co-cultured with PBS serum-treated TAMs when exposed to the same concentration of paclitaxel (Figure 1C), indicating QL serum-treated TAMs decreased the paclitaxel resistance in PC-3-TxR. Correspondingly, QL treatment resulted in significantly reduced IC_50_ of both DU145-TxR and PC-3-TxR to paclitaxel (Figure 1D).

### QL serum reversed the phenotype of TAMs

**Figure 2:**
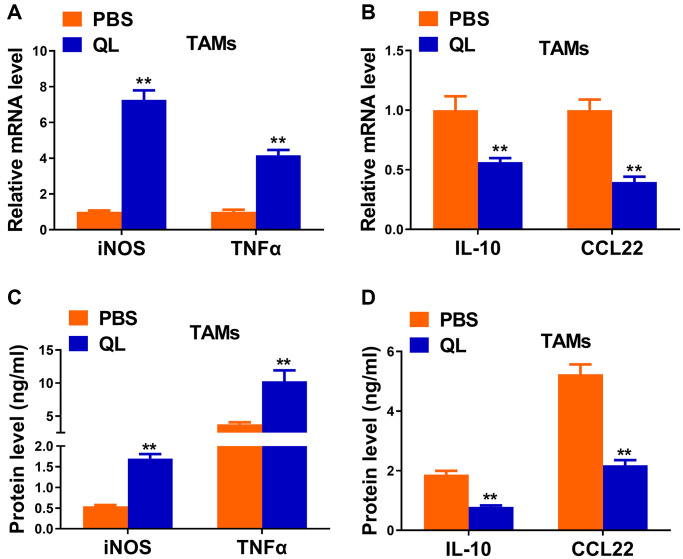
**Qi Ling serum reversed the functional phenotype of TAMs in tumor milieu.** (**A** and **B**) mRNA levels of M1 markers (iNOS and TNFα) and M2 markers (IL-10 and CCL22) in TAMs treated with Qi Ling or PBS were determined by qRT-PCR. (**C** and **D**) Protein levels of M1 markers (iNOS and TNFα) and M2 markers (IL-10 and CCL22) in TAMs treated with Qi Ling or PBS were measured by ELISA. The data represent the mean ± SD. ^**^p < 0.01 determined by Student’s *t*-test.

We continued to evaluate the effects of QL serum on phenotypes of TAMs by measuring the expression of typical M1and M2 markers. QL serum-treated TAMs had significantly increased mRNA expression of iNOS and TNF-α, two M1 markers (Figure 2A). In contrast, the mRNA level of M2 markers IL-10 and CCL22 was significantly decreased in QL serum-treated TAMs (Figure 2B). Correspondingly, significantly increased protein levels of iNOS and TNF-α (Figure 2C), and significantly decreased protein levels of IL-10 and CCL22 (Figure 2D) were detected in TAMs treated with QL serum. Collectively, these results indicated that QL serum converted the M2 phenotype of TAMs to M1 phenotype.

### QL serum suppressed IL-6/STAT3 signaling in TAMs

**Figure 3:**
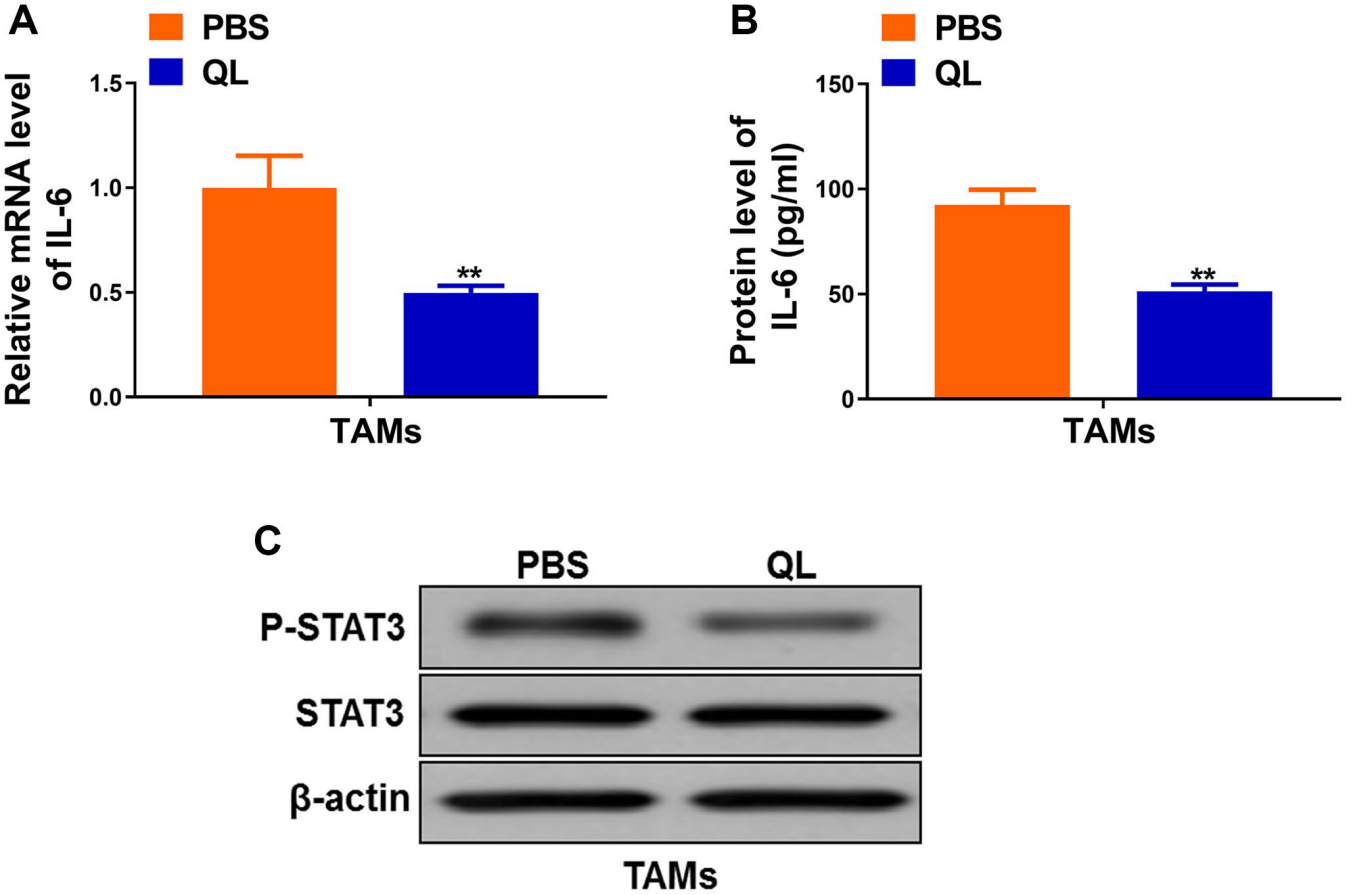
**Qi Ling serum suppressed IL-6/STAT3 signaling in TAMs.** (**A**) mRNA level of IL-6 in TAMs treated with Qi Ling or PBS were determined by qRT-PCR. (**B**) Protein level of IL-6 in TAMs treated with Qi Ling or PBS were measured by ELISA. (**C**) Total STAT3 and p-STAT3 protein levels TAMs treated with Qi Ling or PBS were determined by Western blot. The data represent the mean ± SD. ^**^p < 0.01 determined by Student’s *t*-test.

IL-6/STAT3 signaling pathway has been shown to regulate TAMs polarization in tumor [11, 12]. Therefore, we continued to evaluate the effects of QL serum on IL-6/STAT3 signaling pathway in TAMs. We detected significantly decreased mRNA level (Figure 3A) as well as protein level (Figure 3B) of IL-6 in QL serum-treated TAMs when compared to PBS-treated TAMs. We also detected markedly decreased phosphorylation of STAT3 in QL serum-treated TAMs (Figure 3C). These results demonstrated that QL serum inhibited IL-6/STAT3 signaling pathways in TAMs.

### IL-6 supplement reverted QL serum-induced phenotype transition of TAMs in tumor milieu

**Figure 4:**
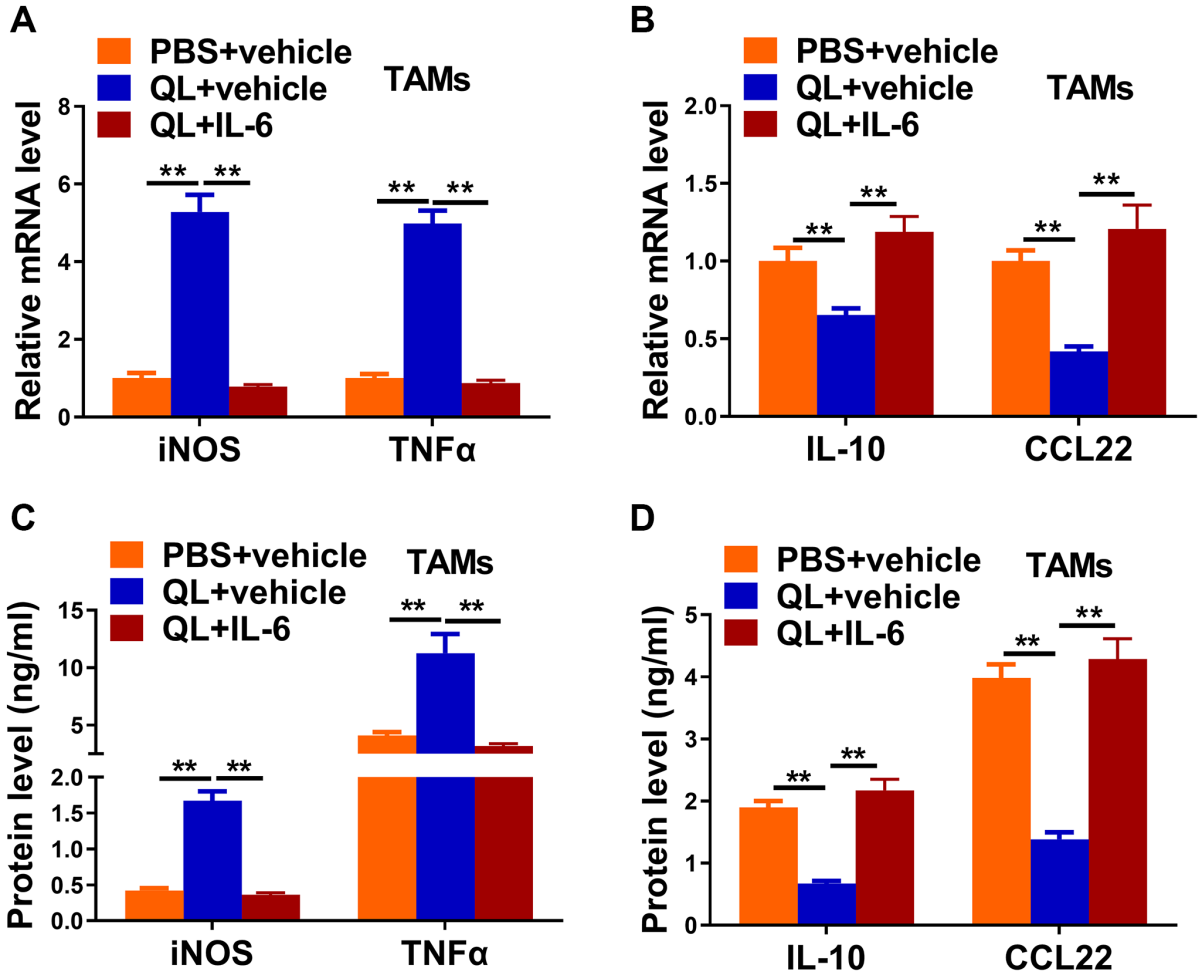
**IL-6 supplement reverted Qi Ling serum-induced phenotype transition of TAMs in tumor milieu.** In the same co-culture system above: TAMs were treated with PBS+vehicle, Qi Ling+vehicle or Qi Ling+IL-6. (**A** and **B**) mRNA levels of M1 markers (iNOS and TNFα) and M2 markers (IL-10 and CCL22) in TAMs of each group were determined by qRT-PCR. (**C** and **D**) Protein levels of M1 markers (iNOS and TNFα) and M2 markers (IL-10 and CCL22) in TAMs of each group were measured by ELISA. The data represent the mean ± SD. ^**^p < 0.01 determined by Student’s *t*-test.

As QL serum targeted IL-6 and promoted the M1 phenotype of TAMs, we wanted to determine whether administration of IL-6 would affect QL serum’s effects. We administrated IL-6 together with QL serum to TAMs and measured the expression of M1, M2 markers. QL serum treatment significantly increased the mRNA expression of M1 makers iNOS and TNF-α, while the upregulation was abolished in the presence of IL-6 (Figure 4A). In contrast, QL serum treatment significantly downregulated the mRNA level of M2 markers IL-10 and CCL22 while IL-6 rescued the mRNA expression of IL-10 and CCL22 in QL serum-treated TAMs (Figure 4B). Correspondingly, IL-6 significantly decreased protein levels of iNOS and TNF-α (Figure 4C), while significantly increased protein levels IL-10 and CCL22 (Figure 4D) in QL-treated TAMs.

### QL serum resensitized paclitaxel-resistant prostate cancer cells to paclitaxel via IL-6/STAT3 signaling in TAMs

**Figure 5:**
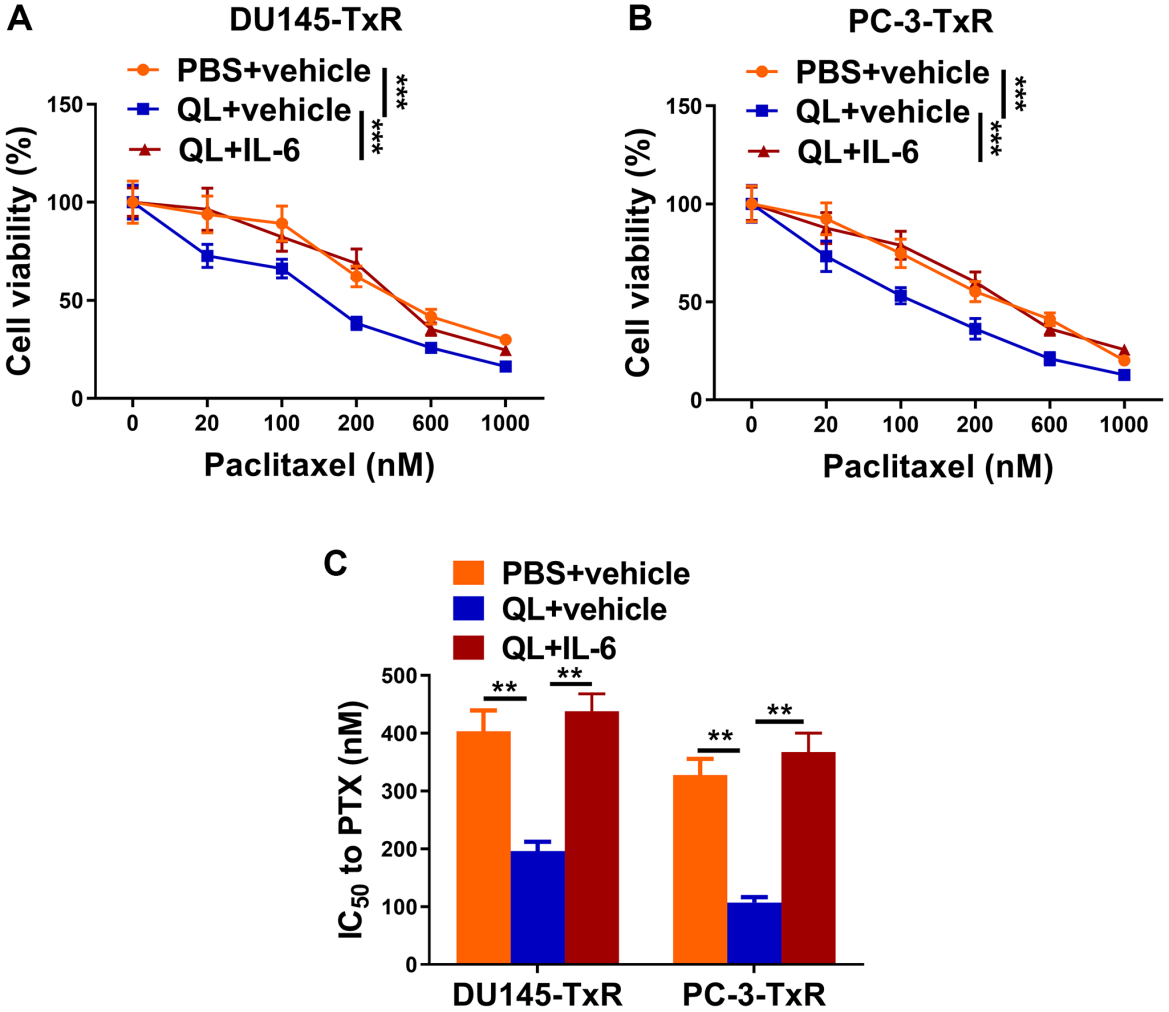
**Qi Ling serum resensitized paclitaxel-resistant prostate cancer cells to paclitaxel via IL-6/STAT3 signaling in TAMs.** In the same co-culture system above: TAMs were treated with PBS+vehicle, Qi Ling+vehicle or Qi Ling+IL-6. (**A** and **B**) MTT assay showed viability of DU145-TxR and PC-3-TxR cells of each group exposed with indicated concentrations of paclitaxel for 24 h. (**C**) IC50 values of DU145-TxR and PC-3-TxR cells of each group were determined from the viability versus paclitaxel concentration curves. The data represent the mean ± SD. ^**^p < 0.01; ^***^p < 0.001 determined by two-way ANOVA followed a post hoc test for panels (**A** and **B**), one-way ANOVA followed a post hoc test for panel (**C**).

Finally, we evaluated the effects of IL-6 and QL serum on sensitivity of paclitaxel-resistant prostate cancer cells to paclitaxel. We treated TAMs with QL serum or QL serum together with IL-6 (QL+IL-6), and measured the viability of co-cultured DU145-TxR or PC-3-TxR cell which were treated with different concentrations of paclitaxel. As shown in Figure 5A, the DU145-TxR cells co-cultured with QL serum-treated TAMs had significantly decreased viability compared to DU145-TxR cells co-cultured with PBS serum-treated TAMs, when treated with same concentration of paclitaxel, indicating QL-treated TAMs decreased paclitaxel-resistance in DU145-TxR. In contrast, the DU145-TxR cells co-cultured with QL serum/IL-6-treated TAMs had significantly increased viability compared to DU145-TxR cells co-cultured with QL-treated TAMs, indicating IL-6 abolished the effects of QL serum on paclitaxel resistance in DU145-TxR (Figure 5B). Correspondingly, QL serum treatment resulted in significantly reduced IC_50_ of both DU145-TxR and PC-3-TxR to paclitaxel while IL-6 resensitized the paclitaxel resistance (Figure 5C). Collectively, these results showed that QL serum resensitized paclitaxel-resistant prostate cancer cells to paclitaxel via IL-6/STAT3 signaling in TAMs.

## DISCUSSION

In present study, we evaluated the potential effects of traditional Chinese medicine QL on paclitaxel resistance in the human prostate cancer. Using the TAMs/prostate cancer cells co-culture system, we identified that QL serum-treated TAMs resulted in decreased paclitaxel resistance in paclitaxel resistant prostate cancer cells DU145-TxR and PC-3-TxR. QL promoted the expression of M1 markers iNOS and TNF-α while inhibited the expression of M2 markers IL-10 and CCL22 in TAMs. QL serum treatment decreased the IL-6 expression and suppressed the activation of STAT3. IL-6 promoted the expression of IL-10 and CCL22 and suppressed the expression of iNOS and TNF-α in TAMs, and diminished the effects of QL serum on paclitaxel resistance.

TAMs have been shown to promote tumor growth and enhance the resistance of cancer cells to both chemotherapy and radiotherapy [8]. In solid tumor, the TAMs present a M2 phenotype and mediate the resistance to chemotherapy by preventing cytotoxic effects of chemotherapy [13]. In present study, we demonstrated that TAMs had high expression of M2 markers IL-10 and CCL22, confirming the M2 phenotype of TAMs in our system. Blocking the M2 phenotype and re-polarizing the M2 macrophage phenotype to the M1 phenotype have been shown to be promising approaches to treat cancer. For example, Dong and colleagues described that fenretinide (4-HPR) significantly suppressed IL-4/IL-13 induced M2 polarization, resulting in fewer M2 macrophages in tumor tissues and dramatically decreased tumorigenesis [14]. CpG-ODN up-regulated the expression M1 markers and effectors in TAMs while decreased the expression of M2 effectors, and induced the anti-tumor effects in mice with established B16 melanoma [15]. We also demonstrated that QL serum treatment promoted the expression of M1 makers including iNOS and TNF-α, while inhibited the expression of M2 markers IL-10 and CCL22. QL serum reprogramed the M2 phenotype to the M1 phenotype, which contributed to the decreased paclitaxel resistance in paclitaxel-resistant prostate cancer cells.

The IL-6/STAT3 signaling pathway is responsible for M2 polarization in cancer [16]. Sorafenib, a tyrosine kinase inhibitor, inhibits STAT3 in macrophages, resulting in restoring the IL-12 production and suppressing the expression of IL-10 [17]. Resveratrol has been shown to decrease the activity of STAT3, suppress M2 polarization of TAMs, and inhibit lung cancer growth [18]. In present study, we found QL serum down-regulated IL-6 expression in TAMs and inhibited the activation of STAT3, which was correlated to the polarization of M1 phenotype in TAMs after QL serum treatment. The QL serum-induced M1 polarization of TAMs resulted in the decreased paclitaxel resistance in paclitaxel-resistant prostate cancer cells. We further demonstrated that these effects of QL serum were abolished when IL-6 was applied to the TAMs, indicating that QL affect TAMs phenotype by targeting IL-6/STAT3 signaling pathway.

In present study, we utilized the TAMs/cancer cells co-culture system and proved that QL serum re-programmed the TAMs to M2 phenotype by targeting IL-6/STAT3 signaling pathway, resulting in restoring the sensitivity of paclitaxel-resistant prostate cancer cells to paclitaxel. Our findings strongly suggested that QL could be used as a therapeutic agent to treat prostate cancer. However, in present study, we utilized serum from QL-treated rats as the reagent for our study. QL treatment significantly increased TNF-α while decreased IL-6 level in serum. TNF-α has been shown to counterbalance the Emergence of M2 Tumor Macrophages by promoting M1 polarization. Therefore, the elevated TNF-α in QL serum should contribute to the regulation of macrophage polarization. Similarly, the decreased IL-6 in QL serum should contribute to the decreased STAT3 activation. Although we identified the difference of TNF-α and IL-6 between QL serum and PBS serum, the difference of other serum components remains unknown. It is useful to analyze the respective compounds and active metabolites in serum since these components may contribute to the effects on macrophages polarization. In addition, it is necessary to evaluate the effects of QL directly using animal model and determine whether QL could re-program TAMs to M1 phenotype *in vivo*. Moreover, current evidences cannot deny the possibility of the involvement of other signaling pathways except for the IL6/STAT3 signaling pathway. Besides IL-6/STAT3 signaling pathway, IL-4/STAT6 is another signaling pathway responsible for M2 polarization [19]. It should be useful to evaluate the effects of QL on IL-4/STAT6 signaling pathway in TAMs.

## CONCLUSIONS

In summary, our findings suggest that QL treatment re-programed the M2 phonotype of TAMs to M1 phenotype and resulted in decreased paclitaxel resistance in the human prostate cancer cells.

## Supplementary Materials

Supplementary Figure 1
