# Supplementary material for: Qi Ling decreases paclitaxel resistance in the human prostate cancer by reversing tumor-associated macrophages function
Source: Aging (Albany NY). 2022 Feb 22;14(4):1812–21. doi: 10.18632/aging.203904 (PMC8908933; doi:10.18632/aging.203904)
Supplement: Supplementary Figure 1 [file aging-14-203904-s001.pdf]

SUPPLEMENTARY FIGURE

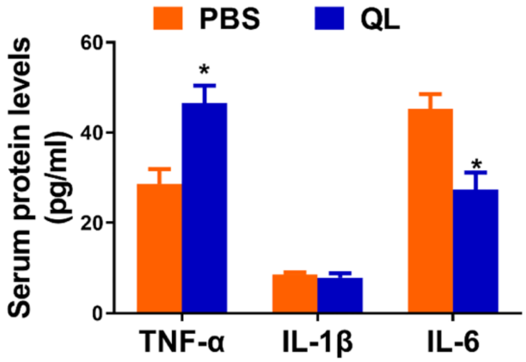

Supplementary Figure 1. TNF-α, IL-1β and IL-6 level in the serum of Qi Ling-treated and PBS-treated rats were measured by ELISA. $^{*}p < 0.05$.
